# Firing Frequency Maxima of Fast-Spiking Neurons in Human, Monkey, and Mouse Neocortex

**DOI:** 10.3389/fncel.2016.00239

**Published:** 2016-10-18

**Authors:** Bo Wang, Wei Ke, Jing Guang, Guang Chen, Luping Yin, Suixin Deng, Quansheng He, Yaping Liu, Ting He, Rui Zheng, Yanbo Jiang, Xiaoxue Zhang, Tianfu Li, Guoming Luan, Haidong D. Lu, Mingsha Zhang, Xiaohui Zhang, Yousheng Shu

**Affiliations:** ^1^State Key Laboratory of Cognitive Neuroscience and Learning, IDG/McGovern Institute for Brain Research, School of Brain and Cognitive Sciences, The Collaborative Innovation Center for Brain Science, Beijing Normal UniversityBeijing, China; ^2^Institute of Neuroscience and State Key Laboratory of Neuroscience, Shanghai Institutes for Biological Sciences, Chinese Academy of Sciences, and University of Chinese Academy of SciencesShanghai, China; ^3^Department of Neurosurgery, Brain Institute, and Department of Neurology, Epilepsy Center, Beijing Sanbo Brain Hospital, Capital Medical UniversityBeijing, China

**Keywords:** firing frequency, fast-spiking neuron, human, monkey, neocortex

## Abstract

Cortical fast-spiking (FS) neurons generate high-frequency action potentials (APs) without apparent frequency accommodation, thus providing fast and precise inhibition. However, the maximal firing frequency that they can reach, particularly in primate neocortex, remains unclear. Here, by recording in human, monkey, and mouse neocortical slices, we revealed that FS neurons in human association cortices (mostly temporal) could generate APs at a maximal mean frequency (F_mean_) of 338 Hz and a maximal instantaneous frequency (F_inst_) of 453 Hz, and they increase with age. The maximal firing frequency of FS neurons in the association cortices (frontal and temporal) of monkey was even higher (F_mean_ 450 Hz, F_inst_ 611 Hz), whereas in the association cortex (entorhinal) of mouse it was much lower (F_mean_ 215 Hz, F_inst_ 342 Hz). Moreover, FS neurons in mouse primary visual cortex (V1) could fire at higher frequencies (F_mean_ 415 Hz, F_inst_ 582 Hz) than those in association cortex. We further validated our *in vitro* data by examining spikes of putative FS neurons in behaving monkey and mouse. Together, our results demonstrate that the maximal firing frequency of FS neurons varies between species and cortical areas.

## Introduction

Neocortical fast-spiking (FS) neurons have been well-identified on the basis of their highly uniform electrophysiological characteristics, including the narrowest action potential (AP) among cortical neurons, fast membrane passive kinetics and their ability to generate high-frequency AP trains with very little frequency accommodation (McCormick et al., 1985; Connors and Gutnick, 1990), therefore also known as the non-accommodating neurons. One specific type of voltage-gated K^+^ channel with fast gating kinetics, the K_v_3 family, has been demonstrated to be sufficient and necessary for the FS phenotype (Erisir et al., 1999; Rudy and McBain, 2001; Lien and Jonas, 2003). Besides, some other passive and active properties of neuronal membrane also contribute to the fast generation and propagation of electric signals in FS neurons (Hu and Jonas, 2014), including postsynaptic dendritic characteristics (Thomson et al., 1993; Geiger et al., 1997; Galarreta and Hestrin, 2001; Goldberg et al., 2003; Lorincz and Nusser, 2008; Hu et al., 2010), coordination of axonal Na^+^ and K^+^ currents (Ogiwara et al., 2007; Lorincz and Nusser, 2008; Hu and Jonas, 2014), and even presynaptic releasing machinery (Xu et al., 2007; De-May and Ali, 2013).

FS neurons are the most abundant cell type among neocortical inhibitory interneurons, composing about one-third to 40% of the neocortical interneuron population (Kawaguchi and Kubota, 1997; Gonchar et al., 2007), thus dominating the regulation of principle neuron activities. The feedforward inhibition mediated by FS neurons is responsible for the temporal precision of postsynaptic APs by limiting the time window of EPSP integration (Pouille and Scanziani, 2001), and expanding the dynamic range of neural circuits by gain control (Pouille et al., 2009; Atallah et al., 2012; Wilson et al., 2012). Besides, FS neurons control the spatial and temporal precision of sensory information processing, by providing cortical feedback and lateral inhibition (Cardin et al., 2009; Couey et al., 2013).

All these physiological functions of FS neurons depend on the reliable generation and propagation of ultra-fast APs (Hu and Jonas, 2014). One way to evaluate the rate of AP generation in these cells is to measure the highest firing frequency. Despite some variance in the intrinsic physiological parameters (Povysheva et al., 2008, 2013), the FS phenotype is conserved across species (Foehring et al., 1991; Krimer and Goldman-Rakic, 2001; Krimer et al., 2005). Impairment of the generation of high-frequency APs in FS neurons will cause severe dysfunction of the neocortex (Lewis et al., 2005; Li et al., 2012), suggesting a vital role of high-frequency firing in FS neurons in the operation of the cortex. It has long been vaguely assumed that the firing frequency of FS neurons could reach 500-600 Hz, or even higher; however, the exact firing-frequency maximum is still unclear, particularly for FS neurons in primate neocortex. In this study, we performed whole-cell recordings from human, monkey, and mouse FS neurons in neocortical slices to investigate their maximal firing frequency and compare their electrophysiological properties across different species. Furthermore, we also investigated the maximal firing frequency of FS neurons in behaving monkey and mouse.

## Materials and methods

The use of human brain tissues was approved by the Biomedical Research Ethics Committee of Shanghai Institutes for Biological Sciences (License No. ER-SIBS-221004). All procedures regarding animal experiments were approved by the Institutional Animal Care and Use Committee of Beijing Normal University.

### Brain tissues for slice recording

The essential information of all the human patients involved in this study is listed in Table 1. In summary, of all the 24 patients involved, 22 were suffering intractable epilepsy and the other 2 were suffering brain tumor without diagnostic epilepsy. The age of the patients ranged from 6 to 60 years. The surgery removal areas included temporal lobe, frontal lobe, occipital lobe, and parietal lobe. Frontotemporal cortical tissues from two adult rhesus monkeys were also used in this study. Tissues sampling was performed when the monkeys were anesthetized with isoflurane (2.5%) before overdosed with sodium pentobarbital and fixed with PFA. An approximately 2 × 2-cm sized block of the cortex was resected from the adjacent area of lateral frontal cortex and superior temporal gyrus. Mouse cortical slices were prepared coronally from the temporal parahippocampal region (mostly entorhinal cortex, Ent. *N* = 4 animals) or primary visual cortex (V1. *N* = 5 animals) of 6-to-8-week-old C57 mice.

**Table 1:** **Patient information**.

**No**.	**Age (year), sex, side**	**Duration (year)**	**Possible precipitation event**	**Seizure type**	**Seizure frequency (times/week)**	**Status epilepsy**	**Surgery removal area**
**Epileptic**							
1	24, M, L	12	Unknown	PC	~1	No	Anterior temporal lobe
2	6, M, R	6	Cyst	PC, gen	0.5-1	No	Anterior temporal lobe
3	53, M, L	32	Unknown	PC, gen	1-2	No	Anterior temporal lobe
4	22, M, L	14	Unknown	PC	2-3	No	Anterior temporal lobe
5	15, M, R	6	Unknown	PC	0.5-1	No	Medial frontal gyrus
6	13, F, L	0.1	Glioma	PC	>30	No	Anterior temporal lobe
7	20, F, R	3	Glioma	PC	~1	No	Middle temporal gyrus
8	22, F, R	18	Unknown	PC	1.5-2.5	No	Anterior temporal lobe
9	44, F, R	26	Unknown	PC, gen	1-1.5	No	Anterior temporal lobe
10	13, F, R	1	Oligodendroglioma	PC, gen	0.5-0.7	No	Anterior temporal lobe
11	40, M, R	21	Unknown	PC	0.5-1	No	Anterior temporal lobe
12	17, M, R	6	Cyst	PC, gen	0.1-0.2	No	Anterior temporal lobe
13	15, M, R	5	Gliosis	PC	1-1.5	No	Anterior temporal lobe
14	30, F, R	15	Unknown	PC, gen	1-1.5	No	Anterior temporal lobe
15	28, M, R	9	Unknown	PC	0.25-1	No	Anterior temporal lobe
16	26, M, R	6	Cavernous hemangioma	PC, gen	0.5-1	No	Anterior temporal lobe
17	20, F, L	8	Unknown	PC	0.2-15	No	Central part of occipital lobe
18	25, M, R	16	Unknown	PC, gen	0.5-1	No	Anterior temporal lobe
19	24, M, R	17	Dysembryoplastic neuroepithelial tumor	PC, gen	1-10	No	Middle temporal gyrus
20	31, F, L	29	Fever	PC	1-1.5	No	Anterior temporal lobe
21	35, F, L	6	Cyst	PC	0.5-1.5	No	Inferior temporal lobe
22	15, M, R	10	Unknown	PC, gen	~0.25	Yes	Parietal lobe
**Non-epileptic**			**Disease**				
23	60, M, L	0.2	Hige-grade glioma in the junction between frontal and parietal lobes				Anterior parietal lobe
24	57, F, R	10	Neurilemmona in the middle cranial fossa				Anterior temporal lobe

*PC, partial complex. PC, gen, partial complex sometimes general*.

### *In vitro* electrophysiology

Neocortical slice preparation and whole-cell patch-clamp recording procedures were performed as previously described (Wang et al., 2015). In brief, human, monkey and mouse cortical tissues were immediately kept in ice-cold oxygenated sucrose artificial cerebrospinal fluid (sucrose-ACSF, in which NaCl was substituted with equiosmolar sucrose and the dextrose was reduced to 10 mM) after isolation from the brain during surgery or under anesthesia (sodium pentobarbital). Slices with a thickness of 250-350 μm were cut using a vibratome (VT1200S, Leica), and incubated at 34.5°C in normal ACSF (in mM: 126 NaCl, 2.5 KCl, 2 MgSO_4_, 2 CaCl_2_, 26 NaHCO_3_, 1.25 NaH_2_PO_4_ and 25 dextrose; 315 mOsm, pH 7.4) for 40 min, and then at room temperature until use. During recordings, slices were perfused with normal ACSF or modified ACSF (modified from normal ACSF, in mM: 3.5 KCl, 1 CaCl_2_, and 1 MgSO_4_) at 35–36°C. Data acquired using normal or modified ACSF were pooled together because we found no statistical difference in any of the electrophysiological parameters between them. Whole-cell pipettes were filled with solution containing (in mM): 140 K-gluconate, 3 KCl, 2 MgCl_2_, 10 HEPES, 0.2 EGTA, 2 Na_2_ATP and 0.2% biocytin (285 mOsm, pH 7.3). Recorded cells resided from cortical layer II to layer V.

Data analysis was carried out off-line. Liquid junction potential (~15 mV) was not corrected. FS neurons were identified by their narrow APs and little steady-state firing-frequency accommodation. We recorded the membrane potential (*V*_m_) responses to a serial of hyperpolarizing and depolarizing current steps (500 ms in duration). In order to push AP firing to its maximal frequency, incremental depolarizing current steps (increment, 50-100 pA) were repetitively applied until a severe decrease in AP amplitudes (smaller than 30 mV, measured as the voltage difference between AP peak and the inter-spike valley) was observed. Since short somatic APs with severe sodium channel inactivation could propagate to distal axonal compartments (Shu et al., 2007) and 30-mV somatic APs could evoke postsynaptic responses (data not shown), we considered those with amplitude >30 mV as full APs. Spikelets with amplitude smaller than 30 mV were not analyzed in this study. The inter-spike interval (ISI) was determined by the time difference between adjacent AP peaks. The mean frequency of APs of each 500-ms step was calculated by dividing the number of APs by the time period of 500 ms, and the maximal mean frequency (F_mean_) is the highest one. The maximal instantaneous frequency (F_inst_) is the inverse value of the minimal ISI observed.

All the physiological values are presented as mean ± s.e.m. in the text. Both the formats of median (inter-quartile range, IQR) and mean ± s.e.m. are presented in the table. Statistical significance of difference between samples was first tested using Kolmogorov-Smirnov (K-S) test. When a *p*-value smaller than 0.05 was found in K-S test, Wilcoxon rank sum (WRS) test was further performed to test the difference in median values. Box-whisker plots in figures are drawn as the Tukey box plot style, in which the bottom and top of the box are the first and third quartiles, and the band inside the box is the second quartile (the median). The lower end of the whisker is the lowest datum that still within 1.5 IQR of the lower quartile, and the higher end of whisker is the highest datum still within 1.5 IQR of the upper quartile. The maximal and/or minimal datum that is not included between the whiskers is plotted as an outlier with a red circle. Cross correlation and calculation of Pearson's correlation coefficient were performed using MATLAB.

### *In vivo* electrophysiology

Single-unit recordings were performed, using tungsten microelectrodes (tip diameter ~50 μm; resistance 0.6–1.3 MΩ; depth 1.6–10.2 mm.), in the right posterior parietal cortex (intraparietal sulcus) of a head-fixed rhesus monkey (male adult), when he was maintaining fixation at a target in the center of the screen in a dark environment. The distance between monkey's eye and the screen was 57 cm. To ensure the fixation, the monkey's eye position was monitored by an eye tracker (Crist Instruments) and compared to the location of fixation target by REX system (NIH). Single units were sorted by an online sorting method (AlphaLab SnR) and re-checked by another spike-sorting method offline (Spike2, CED). The distribution of spike width (time from the trough to the peak) of single units showed clear bimodal distribution. Therefore, we classified them as narrow-spiking units (<0.3 ms) or broad-spiking units (>0.3 ms). For each narrow-spiking unit, instantaneous frequencies were calculated as the reciprocal of ISIs.

*In vivo* extracellular recording from the primary visual cortex of head-fixed awake mice was performed as previously described (Chen et al., 2015). In brief, we placed a custom-made electrode array (tip diameter 25–33 μm; resistance 0.3–0.8 MΩ; depth 300–600 μm) within the binocular area of primary visual cortex. For the spike detection, the threshold was set at about 6 times of the noise level. Raw spikes were sorted as putative single units by using commercial software (Offline Sorter, Plexon) and then classified (K-means method) as broad- and narrow-spiking units based on their waveforms. Visual stimuli were generated with a PC computer and displayed on a CRT monitor placed ~20 cm in front of the animal and centered on its midline. We calculated the spiking responses to the standard sinusoidal drifting gratings (stimulation duration: 2 s, spatial frequency: 0.04 cycles/degree, temporal frequency: 3 Hz) and the distribution of ISIs and F_inst_.

## Results

### Human neocortical FS neurons

**Figure 1:**
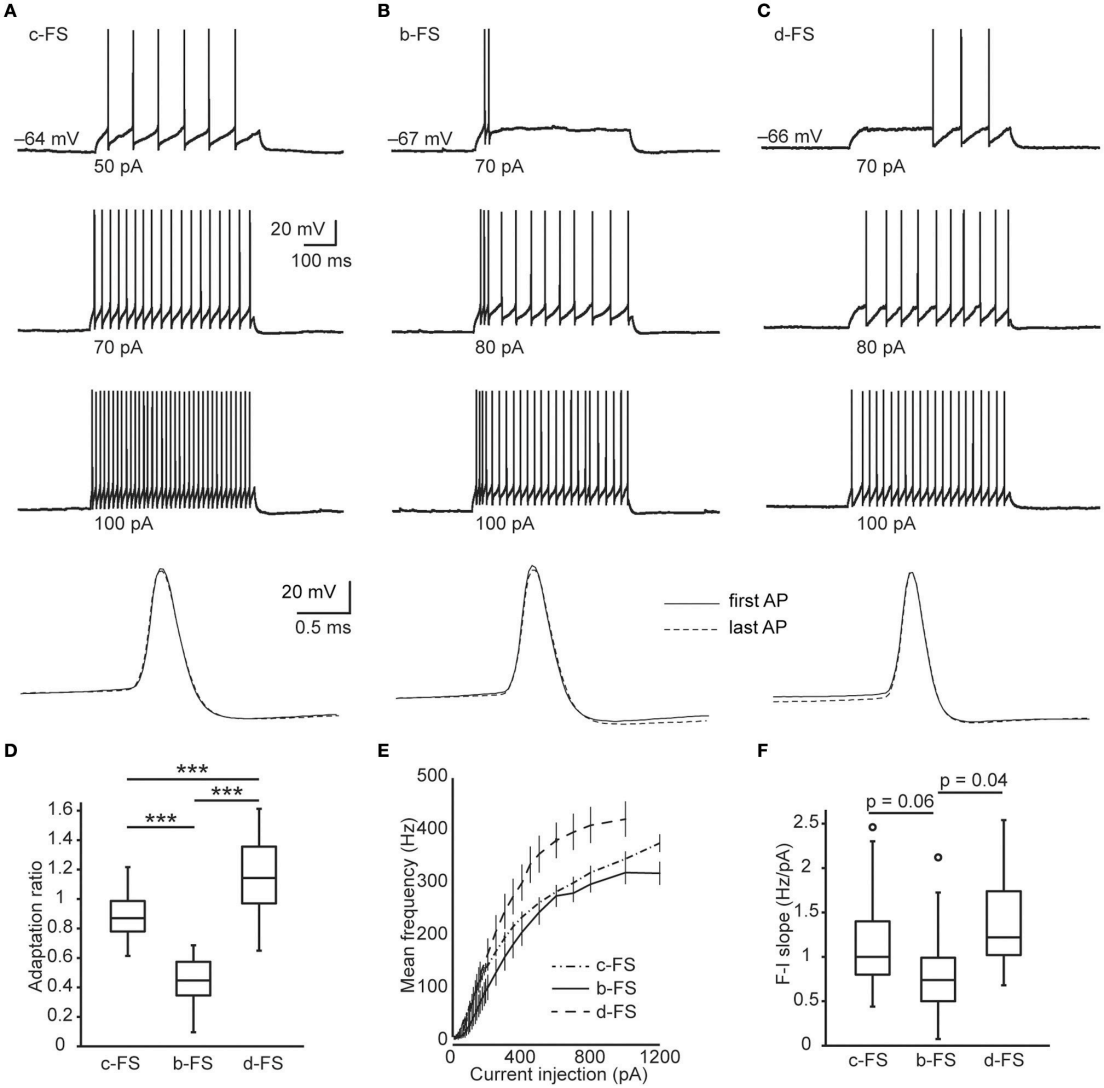
**Subtypes of human neocortical FS neurons. (A–C)** Example *V*_m_ traces from c-FS **(A)**, b-FS **(B)**, and d-FS **(C)** neurons in response to supra-threshold current steps (500 ms in duration). Bottom traces are expanded view of the first (solid line) and last (dashed line) APs in a train and are in the same scale. **(D)** Comparison of adaptation ratio between human fast-spiking neuronal types. **(E)** F-I curves of all three types of human fast-spiking neurons. **(F)** Comparison of initial F-I slope between human fast-spiking neuronal types. The circles in box-whisker plots are the maximal outliers. ^***^, *p* < 0.005, Wilcoxon rank sum test.

In human neocortical slices, we tested the AP firing pattern of every recorded neuron by step current injections. FS neurons were identified by their narrow APs (width < 0.4 ms, in average, 0.27 ± 0.01 ms.), slight AP amplitude drop (1st to 2nd AP, 0.15 ± 0.10 mV), and no significant accommodation in AP waveform or steady-state spiking frequency (Figures 1A–C). In total, we collected 92 FS neurons, whose intrinsic physiological parameters were presented in Table 2. These human FS neurons were categorized as three subtypes, in terms of their initial firing patterns in response to threshold current steps. The first subtype fired APs at a uniform instantaneous frequency during the 500-ms stimulating period, termed the classical FS (c-FS) neurons (*n* = 71. Figure 1A). The second subtype fired AP burst at the beginning, termed the bursting FS (b-FS) neurons (*n* = 11. Figure 1B). And the third subtype fired APs after a time delay (much longer than inter-spike intervals), termed the delayed firing FS (d-FS) neurons (*n* = 10. Figure 1C). These three subtypes resemble the electrophysiological subtypes of cortical FS neurons of rodents (Gupta et al., 2000; Wang et al., 2002). Besides the adaptation ratio, which can apparently differentiate those three subtypes (c-FS 0.89 ± 0.02, b-FS 0.44 ± 0.06, and d-FS 1.15 ± 0.10. Figure 1D), a slight difference in the responsiveness to current injection was found between b-FS and d-FS neurons (Figures 1E,F). The initial F-I slope of b-FS neurons (0.86 ± 0.20 Hz/pA) was lower than that of d-FS (1.38 ± 0.21 Hz/pA, *p* = 0.04, WRS test), but not significantly lower than that of c-FS neurons (1.05 ± 0.05 Hz/pA, *p* = 0.06, WRS test. Figure 1F).

**Table 2:** **The intrinsic physiological parameters of cortical FS neurons**.

	**R_in_ (MΩ)**	**τ_m_ (ms)**	**C_m_ (pF)**	**Adaptation ratio**	**AP threshold (mV)**	**AP width (ms)**	**AHP (mV)**	**F_mean_ (Hz)**	**F_inst_ (Hz)**
**ALL HUMAN**									
FS	189.7 ± 12.6	10.0 ± 0.5	63.7 ± 3.1	0.86 ± 0.03	−43.4±0.5	0.27 ± 0.01	19.9 ± 0.4	338.1 ± 9.9	453.4 ± 12.5
(*n* = 92)	158.5(98.4–250.3)	8.3(6.7–12.3)	56.9(41.7–76.5)	0.85(0.73–1.00)	−43.7(−46.2—-41.1)	0.27(0.24–0.30)	19.9(17.0–22.6)	326.9(265.6–378.6)	430(379.8–506.5)
c-FS	207.5 ± 15.2	10.7 ± 0.7	61.6 ± 3.3	0.89 ± 0.02	−43.3±0.5	0.27 ± 0.01	20.2 ± 0.5	327.8 ± 11.3	445.8 ± 14.3
(*n* = 71)	190.2(106.0–266.5)	9.1(7.2–13.6)	55.6(41.2–74.8)	0.86(0.77–0.99)	−43.9(−45.6—-40.6)	0.27(0.24–0.31)	20.0(17.2–23.1)	316.0(257.0–363.6)	427.4(365.5–507.8)
b-FS	117.2 ± 20.5	7.3 ± 0.5	74.3 ± 9.9	0.44 ± 0.06	−44.9±1.1	0.27 ± 0.01	16.5 ± 1.0	335.4 ± 13.9	422.7 ± 12.0
(*n* = 11)	95.0(84.0–132.0)	7.2(6.2–8.2)	65.3(55.0–90.0)	0.45(0.35–0.57)	−44.0(−47.2—-42.6)	0.28(0.26–0.28)	16.8(15.0–18.7)	339.6(317.4–373.6)	423.0(403.6–441.4)
d-FS	143.4 ± 18.4	8.2 ± 0.8	66.0 ± 14.3	1.15 ± 0.10	−42.2±1.3	0.22 ± 0.01	21.0 ± 0.8	414.2 ± 36.4	541.2 ± 47.9
(*n* = 10)	136.5(113.6–152.1)	7.8(6.8–8.3)	54.2(42.0–70.7)	1.14(0.97–1.36)	−42.3(−45.6—-40.5)	0.21(0.19–0.24)	21.6(19.2–22.0)	422.6(365.7–484.5)	534.6(472.0–622.8)
**MONKEY**									
FS	180.6 ± 23.8	8.7 ± 0.6	58.3 ± 4.9	0.82 ± 0.05	−44.3±1.2	0.26 ± 0.01	15.6 ± 0.8	449.6 ± 24.5	611.2 ± 31.3
(*n* = 23)	151.0(123.3–181.0)	8.1(6.8–10.6)	55.1(47.0–69.6)	0.88(0.59–0.97)	−44.5(−48.3—-39.3)	0.24(0.23–0.29)	15.7(12.6–17.2)	445.0(373.8–526.1)	593.5(539.1–692.0)
**MOUSE**									
Ent. FS	179.0 ± 14.4	9.8 ± 0.7	61.7 ± 3.2	1.46 ± 0.41	−34.1±1.1	0.39 ± 0.01	22.8 ± 0.9	215.3 ± 8.6	341.9 ± 11.5
(*n* = 41)	158.3(112.8–219.2)	8.7(7.0–10.7)	57.7(48.2–69.5)	0.90(0.69–1.10)	−33.9 (−39.9—-28.0)	0.40(0.35–0.44)	22.5(20.5–27.0)	206.0(180.0–244.0)	324.7(286.5–396.8)
Mouse V1	108.5 ± 6.0	5.4 ± 0.2	54.5 ± 2.4	1.39 ± 0.22	-−38.9±0.7	0.29 ± 0.01	21.6 ± 0.5	415.4 ± 11.9	581.7 ± 15.0
FS (*n* = 45)	106.6(79.9–129.3)	5.3(4.6–6.2)	52.5(44.0–66.0)	1.01(0.73–1.40)	−38.8(−41.2—-36.0)	0.28(0.26–0.30)	21.8(20.0–23.9)	424.0(372.0–470.0)	586.9(511.2–643.5)

*R_in_, input resistance; τ_m_, membrane time constant; C_m_, membrane capacitance; Ent., entorhinal cortex; V1, primary visual cortex; Data were presented as mean ± s.e.m. and median(IQR)*.

**Figure 2:**
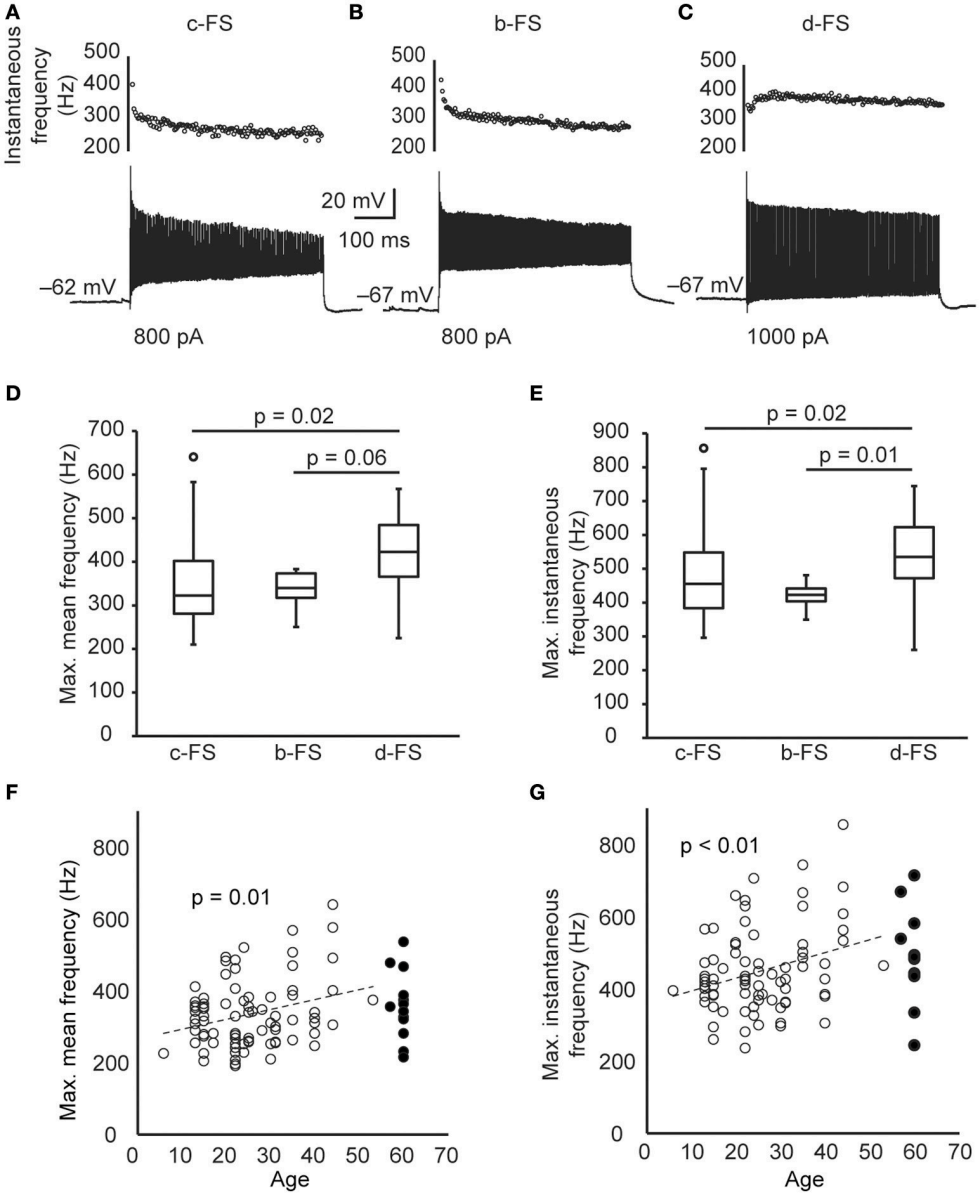
**The maximal firing frequency of human cortical FS neurons. (A–C)** The *V*_m_ responses composing the highest number of APs of the same FS neurons in Figures 1A–C in response to 500-ms current steps. Dot plots are the corresponding instantaneous AP frequency. **(D)** The maximal mean AP frequency induced by 500-ms current steps of human FS neurons. **(E)** The maximal instantaneous AP frequency of human FS neurons. **(F–G)** The scatter plot of the maximal mean frequency **(F)** and the maximal instantaneous frequency **(G)** of human FS neurons vs. patient age. Dashed lines are the linear fit. Open and filled circles are epileptic and non-epileptic neurons, respectively. The circles in box-whisker plots are the maximal or minimal outliers.

In response to 500-ms current steps, the maximal mean AP frequency (F_mean_) without severe inactivation (see Materials and Methods. Figures 2A–C) of all human FS neurons was 338.1 ± 9.9 Hz (ranging from 190.6 to 640.7 Hz) and the maximal instantaneous AP frequency (F_inst_) was 453.4 ± 12.5 Hz (ranging from 236.5 to 856.2 Hz). The d-FS neurons could fire at a slightly higher frequency (F_mean_ = 414.2 ± 36.4 Hz, F_inst_ = 541.2 ± 47.9 Hz) than c-FS (F_mean_ = 327.8 ± 11.3 Hz, *p* = 0.02, F_inst_ = 445.8 ± 14.3 Hz, *p* = 0.02, WRS test) and b-FS neurons (F_inst_ = 422.7 ± 12.0 Hz, *p* = 0.01, but not significant for F_mean_, 335.4 ± 13.9 Hz, *p* = 0.06, WRS test. Figures 2D,E). No significant difference was found between the firing frequency maxima of c-FS and b-FS neurons (F_mean_, *p* = 0.28, F_inst_, *p* = 0.27, K-S test). Those FS neurons with higher F_mean_ also expressed higher F_inst_, with a linear regression equation of y = 1.13x + 70.4, and the Pearson's correlation coefficient was 0.90.

The human cortical tissues that we used were obtained from patients that varied widely across age and disease (Table 1). Specifically, most of the tissue samples experienced repetitive epileptic seizures, which was always accompanied by changes in neuronal excitabilities. Therefore, we next compared the maximal firing frequency of FS neurons between tissues from epileptic (*n* = 79 cells) and non-epileptic patients (*n* = 13 cells), and found no significant difference (F_mean_, 334.6 ± 10.8 vs. 359.8 ± 26.9 Hz, *p* = 0.37; F_inst_, 448.2 ± 13.4 vs. 485.0 ± 36.2 Hz, *p* = 0.06, K-S test.). Considering only epileptic samples, we found that the maximal firing frequency of FS neurons positively correlated with the age of patients (Pearson's correlation coefficient 0.29 and 0.30, *p* = 0.01 and < 0.01 for F_mean_ and F_inst_. Figures 2F,G). This result suggests that the maximal firing frequency of FS neurons increased with age. In the following inter-species comparisons, we chose only the data that were obtained from young adults (>18 and < 40 years old).

**Figure 3:**
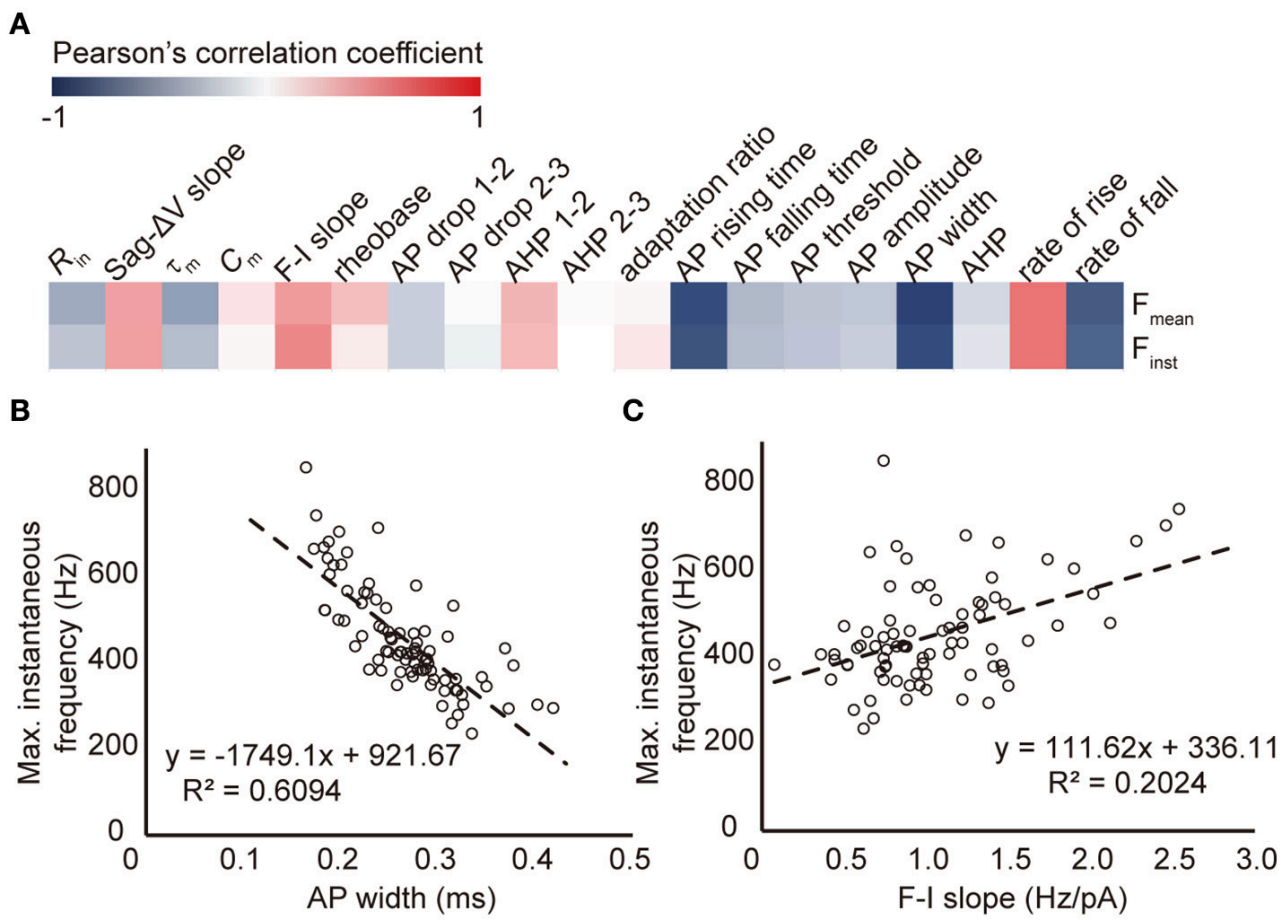
**The correlation between maximal firing frequency and other intrinsic properties of human FS neurons. (A)** The color coded correlation map of all intrinsic parameters examined in human FS neurons. **(B–C)** The linear fit of maximal instantaneous frequency vs. AP width **(B)** and F-I slope **(C)** of all human FS neurons.

It is supposed that higher firing frequency requires faster AP rising and falling, thus narrower AP. We next examined the cross correlation between the maximal firing frequency and neuronal intrinsic properties of FS neurons, including the AP width (Figure 3A). We found that the F_mean_ and F_inst_ were strongly correlated with AP waveform parameters, including AP rising rate and falling rate. Specifically, F_inst_ was negatively and linearly correlated with AP width (measured at the threshold current stimulation), with a regression equation of y = −1749x + 922 and the Pearson's correlation coefficient of −0.78, *p* < 0.005 (Figure 3B). Besides, we also found a positive correlation between the maximal firing frequency and the F-I slope (*p* < 0.005. Figure 3C). In other words, those FS neurons expressing higher responsiveness to current injection (higher initial F-I slope) tended to possess higher maximal firing frequencies.

### Comparison of human FS neurons to those of monkey and mouse

**Figure 4:**
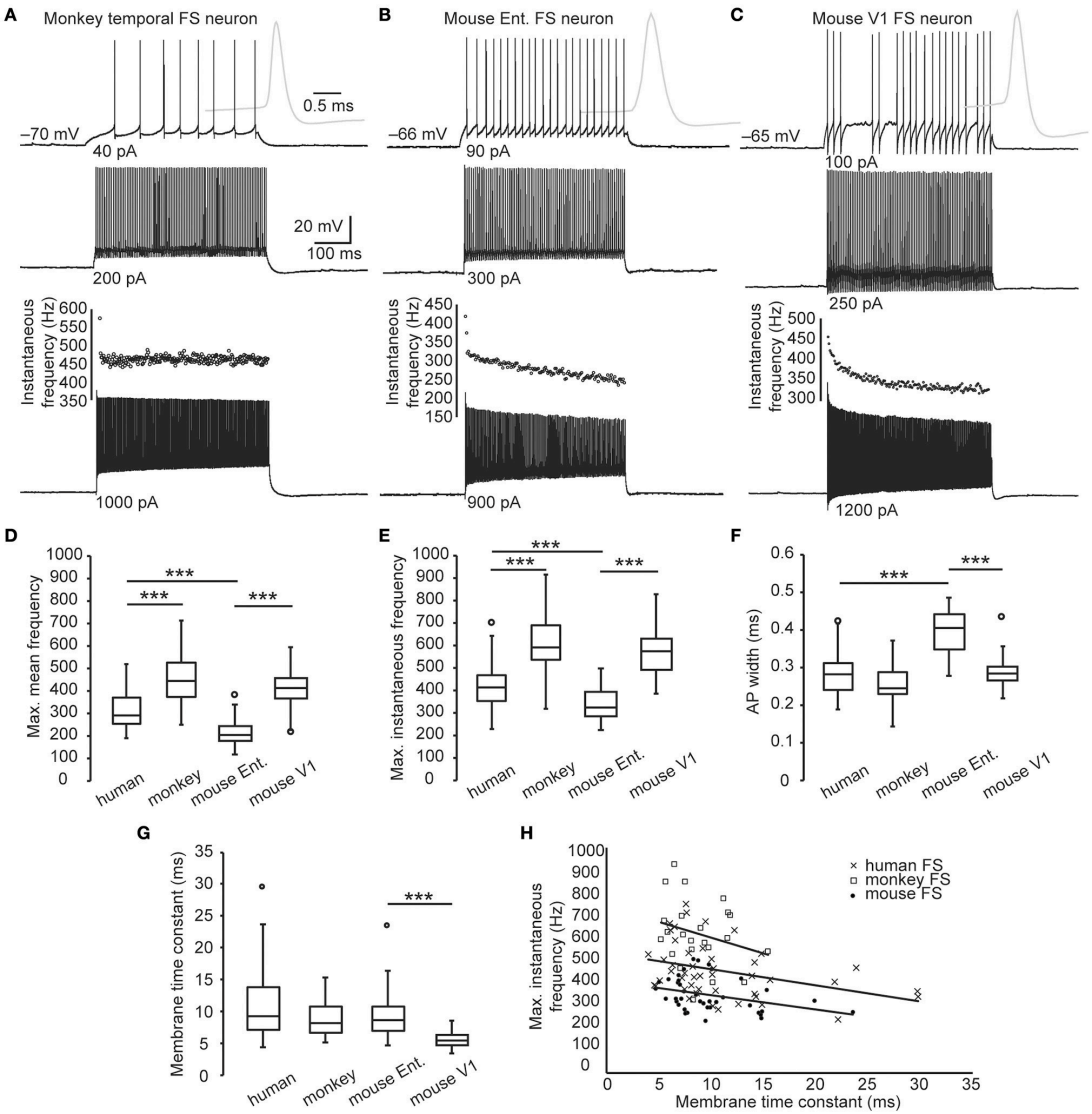
**Maximal firing frequency of monkey and mouse FS neurons. (A–C)** The *V*_m_ traces of FS neurons from monkey **(A)**, mouse entorhinal **(B)**, and V1 cortex **(C)** in response to supra-threshold current steps (500 ms in duration). Gray traces are expanded view of the first APs. Dot plots indicate the corresponding instantaneous AP frequency of the *V*_m_ responses composing the highest number of APs. **(D–E)** Comparison of the maximal firing frequency of monkey, human, and mouse FS neurons. **(F)** Comparison of the AP width between different species. **(G)** Membrane time constants of human, monkey and mouse FS neurons. **(H)** The scatter plot of the maximal instantaneous frequency vs. membrane time constants of each cortical FS neurons from human (cross), monkey (box), and mouse (dot). Solid lines are linear fits for each species. Ent., entorhinal cortex. The circles in box-whisker plots are the maximal or minimal outliers. ^***^, *p* < 0.005, Wilcoxon rank sum test.

Most of the human brain slices involved in this study were derived from association cortices. We next examined the AP firing of FS neurons in the association cortices of monkey (lateral frontal and superior temporal cortex, *n* = 23, Figure 4A), and mouse (entorhinal cortex, *n* = 41. Figure 4B). All of them were young adults (see Materials and Methods). Their intrinsic physiological parameters were presented in Table 2. In response to 500-ms current steps identical to those applied to human neurons, monkey FS neurons fired repetitive APs with a F_mean_ of 449.6 ± 24.5 Hz and a F_inst_ of 611.2 ± 31.3 Hz, both of which were much higher than those of human FS neurons (F_mean_, 332.8 ± 14.6 Hz, F_inst_, 450.0 ± 17.9 Hz, *n* = 46, *p* < 0.005, WRS test. Figures 4D,E); whereas the F_mean_ (215.3 ± 8.6 Hz) and F_inst_ (341.9 ± 11.5 Hz) of mouse entorhinal FS neurons were much lower than those of human (*p* < 0.005, WRS test. Figures 4D,E). We further examined FS neurons in mouse V1 (*n* = 45. Figure 4C), and revealed that the maximal firing frequency of FS neurons in V1 (F_mean_ 415.4 ± 11.9 Hz, F_inst_ 581.7 ± 15.0 Hz) was much higher than those in the entorhinal cortex (*p* < 0.005, WRS test. Figures 4D,E). These results demonstrate that the maximal firing frequency of FS neurons varies between similar cortices of different species and between cortices of the same species.

Comparison of the AP width of FS neurons revealed that they were wider in entorhinal cortex of mouse (0.39 ± 0.01 ms) than in human (0.28 ± 0.01 ms, *p* < 0.005, WRS test.) and in V1 (0.29 ± 0.01 ms, *P* < 0.005, WRS test. Figure 4F). Interestingly, there was no significant difference between the AP width of human and monkey FS neurons (0.27 ± 0.01 vs. 0.26 ± 0.01 ms, *p* = 0.44, K-S test. Figure 4F), though the F_inst_ of monkey FS neurons was also linearly correlated with the AP width (regression equation, y = −2052x + 1136, Pearson's correlation coefficient, −0.80, *p* < 0.005). These results indicated that the difference in maximal firing frequency between human and monkey FS neurons was not accompanied by a discrepancy in the AP width.

FS neurons are not only capable of generating high-frequency APs but also responsive to high-frequency synaptic inputs. Passive properties, such as a smaller membrane time constant, are required for reliable *V*_m_ responses to high-frequency current inputs. Even though we found no significant difference in the membrane time constant of FS neurons between species (human 10.8 ± 0.9 ms, monkey 8.7 ± 0.6 ms, *p* = 0.42, mouse entorhinal 9.8 ± 0.7 ms, *p* = 0.80, K-S test. Figure 4G), negative correlation between F_inst_ and the membrane time constant was discovered in all the three species (Pearson's correlation coefficient, human −0.27, *p* < 0.005, monkey −0.26, *p* = 0.09, mouse -0.35, *p* = 0.006. Figure 4H). This result suggests that, within species, those FS neurons possessing higher maximal firing frequency tended to be more responsive to high-frequency inputs. Furthermore, FS neurons in the mouse V1 expressed a much shorter membrane time constant (5.4 ± 0.2 ms, *p* < 0.005, WRS test), suggesting an even better responsiveness to high-frequency inputs.

### High-frequency firing of FS neurons *in vivo*

**Figure 5:**
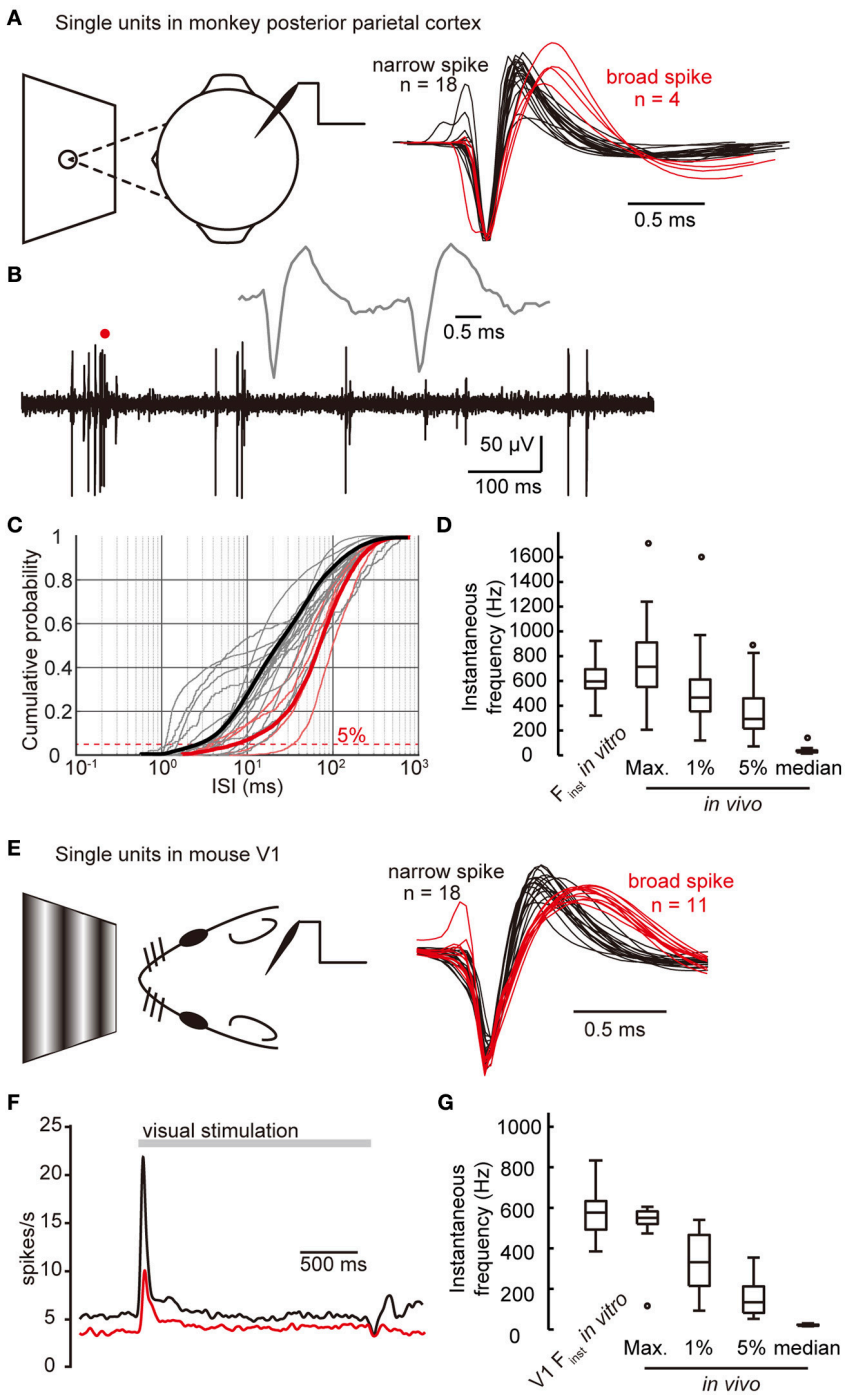
**High-frequency firing of FS neurons ***in vivo***. (A)** Left, schematic drawing shows that single-unit recording is performed in the posterior parietal cortex of a monkey keeping fixation at a target point. Right, example averaged spike waveforms generated from 18 narrow-spiking units and 4 broad-spiking units recorded in monkey parietal cortex. **(B)** Example trace recorded in monkey neocortex during eye fixation period. Insetted gray line is the expanded view of the part indicated by a red dot, showing two neighboring spikes from the same unit, and they are fired at a high instantaneous frequency (~600 Hz). **(C)** Cumulative distribution of ISIs of all monkey units. Gray and light red lines are individual narrow- and broad-spiking units. Thick black and red lines are the cumulative distribution taking all 18 narrow units and all 4 broad units together, respectively. Red dashed line indicates the fifth percentile. X-axis values are in log scale. **(D)** The high-value extent of instantaneous frequency of cortical narrow-spiking units in monkey, along with *in vitro* F_inst_ data for comparison. **(E)** Left, schematic drawing shows that single-unit recording is performed in the primary visual cortex of a mouse receiving visual stimuli. Right, averaged spike waveforms generated from 18 narrow-spiking units (black) and 11 broad-spiking units (red). **(F)** The spike density curves of narrow- (black) and broad- (red) spiking units in mouse V1 in response to visual stimuli. Bin size, 1 ms. **(G)** The high-value extent of instantaneous frequency of cortical narrow-spiking units in mouse. Max., the highest instantaneous frequency; 1 and 5%, the first and fifth percentiles of instantaneous frequency. The circles in box-whisker plots are the maximal or minimal outliers.

Our *in vitro* results were acquired under identical maintenance condition and using the same pattern of stimulation. They revealed the differences in the intrinsic spiking ability of cortical FS neurons between species and between cortices. In order to investigate to what extent the firing frequency of FS neurons could reach under physiological activation, we further examined the firing of FS neurons in the neocortex of behaving monkey and mouse. In one monkey keeping eye fixation at a target point, we performed single-unit recordings in its posterior parietal cortex (Figure 5A), which was supposed to be activated during the period of attention captured “bottom-up” by salient stimuli (Kastner and Ungerleider, 2000; Corbetta and Shulman, 2002). Based on their spike waveforms, we identified 18 narrow-spiking units, which were putatively FS neurons (Barthó et al., 2004; Niell and Stryker, 2010) and 4 broad-spiking units (Figures 5A,B. see also Materials and Methods), and analyzed all their instantaneous spiking frequencies. We plotted the cumulative distribution curves of ISIs for individual single units and found the curves of narrow-spiking units are more left-shifted (i.e., higher firing frequency) than those of broad-spiking units (Figure 5C). The maximal instantaneous frequency of those putative FS neurons was 746.0 ± 85.8 Hz (ranging from 206.7 to 1717.0 Hz.), close to that obtained *in vitro* (611.2 ± 31.3 Hz, *p* = 0.16, K-S test. Figure 5D). Considering that FS neurons do not always fire at their highest frequency during the fixation period, we also examined how often FS neurons fire at high frequencies. With a 1% (or 5%) chance, monkey FS neurons could fire at instantaneous frequencies higher than 542.1 ± 85.7 Hz (or 363.4 ± 59.0 Hz. Figures 5C,D).

Next, we investigated the firing frequency of cortical FS neurons of mice by recording single units in mouse V1 in response to simple visual stimuli (sinusoidal drifting gratings. Figure 5E). During a 2-s stimulation period, narrow-spiking units (putatively FS neurons, Figure 5E) transiently elevated their firing frequency at the onset and fired with higher frequency than broad-spiking units (Figure 5F). By analyzing all the ISIs during the stimulation period of individual single units, we found that narrow-spiking units discharged with a maximal instantaneous frequency of 561.8 ± 13.7 Hz (*n* = 18), close to that obtained *in vitro* (581.7 ± 15.0 Hz, *p* = 0.09, K-S test, *p* = 0.56, WRS test. Figure 5G). With a 1% (or 5%) chance, narrow-spiking units discharged with instantaneous frequencies higher than 437.7 ± 21.0 Hz (or 247.1 ± 23.1 Hz. Figure 5G). Due to lack of a well-established behavior paradigm to activate the association cortices of mouse, we could not validate the difference in maximal firing frequency of FS neurons between cortices by *in vivo* recordings. Together, our results demonstrate that the maximal firing frequency of FS neurons *in vivo* is comparable to that found *in vitro*, and is able to reach near-kilohertz ultrafast frequencies.

## Discussion

In this study, we first described and compared the maximal firing frequencies of FS neurons in human, monkey, and mouse neocortex, and also examined its correlation with different neuronal intrinsic properties. And then, the data about maximal firing frequency obtained in cortical slices of animals were validated by *in vivo* recordings in behaving monkey and mouse. These data are critical for a comprehensive understanding of the physiological characteristics of neocortical neurons, especially in primates. To quantify the maximal firing frequency of FS neurons, here we employed two physiological parameters, the F_mean_ and F_inst_. The F_inst_ evaluates the extreme time precision of the output signal, and the F_mean_ is the average AP frequency across a time period (500 ms in this study). These two parameters correlate with each other. Interestingly, in association cortices, the firing frequency of monkey FS neurons can reach a much higher level than that of human, whereas the maximal firing frequency of mouse FS neurons is much lower than that of primates. These data suggest better time resolution of cortical inhibition possessed by primates than rodents, and even better for monkeys. Higher firing frequency of FS neurons may also ensure the temporal precision of APs in postsynaptic pyramidal cells (Royer et al., 2012) and fine the tuning of network oscillations (Bartos et al., 2007; Cardin et al., 2009). Though we do not have evidence for the behavioral relevance of the higher maximal firing frequency of monkey FS neurons, the resulting increased dynamic range of cortical circuit might be one of the neural basis of the higher capacity of working memory of non-human primates than humans (Inoue and Matsuzawa, 2007). Besides, the finer temporal resolution resulted from faster spiking might contribute to the reaction rate and agility required for the jungle life of monkeys. We should also note that higher firing frequency in inhibitory interneurons could facilitate asynchronous GABA release (Hefft and Jonas, 2005; Jiang et al., 2012), which may cause desynchronization of neural network (Manseau et al., 2010).

Previous studies revealed distinct neuronal intrinsic properties at different age (McCormick and Prince, 1987; Huguenard et al., 1988; Disterhoft and Oh, 2006; Luebke and Chang, 2007). Indeed, we found a positive correlation between the maximal firing frequency and the age of patients, indicating a developmental or age-related change in the spiking machinery of FS neurons (Massengill et al., 1997; Boda et al., 2012). Therefore, to compare with lab animals that are all young adults, we used only the data from patients within the age range from 18 to 40 years. We have to note that, as shown in Table 1, our sample tissues varied greatly including a wide range of age, different cortical regions, and disease types. The etiologic mechanism and duration of illness also differ in epileptic patients. Previous studies have demonstrated a link between Kv3 channels, the most important ion channel for FS phenotype, and epileptic seizure (Lau et al., 2000; Lee et al., 2009). Therefore, it is possible that the difference in maximal firing frequency between human and monkey FS neurons could be attributed to pathological changes in human neurons; however, we found no significant difference in the maximal firing frequency between non-epileptic and epileptic FS neurons. Since it is impossible to have strict control of human samples (age, gender, brain region, disease type etc.) for this comparison, we are not able to rule out the contribution of pathological changes. Considering that animals used in this study did not suffer any epileptic seizure, the difference of maximal firing frequency between primates and mice should not be attributed to any pathological conditions.

Differences in the distribution and biophysical properties of voltage-gated ion channels (Vacher et al., 2008) could contribute to the interspecies difference of firing frequency limit. Besides, neuronal morphological architecture may also be involved (Yáñez et al., 2005; Povysheva et al., 2008). However, obviously evolution cannot explain the differences between FS neurons in V1 and entorhinal cortex of mouse. Cortical FS neurons originate from the same group of progenitors during early developmental stage (Wonders and Anderson, 2006). Therefore, differences in physiology are most likely determined by their postnatal maturation (Massengill et al., 1997; Itami et al., 2007; Goldberg et al., 2011; Yang et al., 2014) and activity-dependent refinement (Miller et al., 2011; Dehorter et al., 2015), endowing FS neurons with distinct roles in different cortical circuits.

Our *in vivo* data and some other investigations in live animals showed that the maximal instantaneous AP frequency of FS neurons was close to or even higher than our *in vitro* data (Nuñez et al., 1993; Azouz et al., 1997; Contreras and Palmer, 2003; Nowak et al., 2003). Discrete synaptic inputs *in vivo*, which better facilitate high-frequency spiking than step current injection, may explain the higher maximal firing frequency of FS neurons *in vivo*. In the primary sensory cortices (such as V1), FS neurons may have to code higher-frequency inputs driven by sensory stimuli, and that may explain why we found a much higher maximal firing frequency and a shorter membrane time constant in FS neurons of mouse V1 than the entorhinal cortex. Passive kinetics of neuronal membrane not only affects the AP firing frequency but also shapes the neuronal response to synaptic inputs. Within species, FS neurons with higher maximal firing frequency tend to express shorter membrane time constant, suggesting possibly better responsiveness to high-frequency inputs. However, there is no difference in the membrane time constant between the three examined species.

Cumulative evidence from morphological and molecular analysis have demonstrated that the FS neurons are a heterogeneous neuronal type, even if the parvalbumin-positive basket cells are the vast majority of cortical FS neurons (Kawaguchi et al., 1987; Wang et al., 2002, 2004; Markram et al., 2004). In addition to the parvalbumin-positive basket neurons, some of the FS neurons are positive for somatostatin, cholecystokinin or vasoactive intestinal peptide-positive, and some of them are differently shaped. We did not further subdivide them into groups, but whether the maximal firing frequency varies between FS subtypes remains to be investigated.

## Author contributions

BW, WK, LY, SD, TH, YL, RZ, YJ, and XXZ performed *in vitro* recordings. HDL helped with monkey tissue processing. TL and GL helped with human tissue processing. BW, WK, QH, and YS analyzed the data. JG and MZ performed monkey *in vivo* recordings. GC and XHZ performed mouse *in vivo* recordings. BW and YS designed the experiments and wrote the paper.

### Conflict of interest statement

The authors declare that the research was conducted in the absence of any commercial or financial relationships that could be construed as a potential conflict of interest.
